# The association among negative life events, alexithymia, and depressive symptoms in a psychosomatic outpatient sample

**DOI:** 10.1186/s12888-024-05902-0

**Published:** 2024-06-18

**Authors:** Yinghan Xie, Dandan Ma, Yanping Duan, Jinya Cao, Jing Wei

**Affiliations:** Department of Psychological Medicine, Peking Union Medical College Hospital, Chinese Academy of Medical Sciences and Peking Union Medical College, Shuaifuyuan 1, Dongcheng District, Beijing, 100730 PR China

**Keywords:** Negative life events, Alexithymia, Depression, Stepwise regression analysis

## Abstract

**Background:**

Depression is a life-threatening mental health problem. Various factors have been demonstrated to be associated with depressive symptoms, including negative life events (NLEs) and alexithymia. A retrospective study was conducted to investigate the relationship among negative life events, alexithymia, and depression symptoms in a psychosomatic outpatient sample in China.

**Methods:**

A total of 2747 outpatients (aged 18 – 65) were included in this investigation. The Life Events Scale (LES), Toronto alexithymia scale (TAS-26), and 9-item Patient Health Questionnaire (PHQ-9) were used to assess NLEs, alexithymia, and depressive symptoms, respectively. A stepwise regression analysis model was established to investigate the relationship among alexithymia, NLEs, and depressive symptoms.

**Results:**

Overall, 67.0% of the patient sample had a PHQ-9 score of 10 or higher. The stepwise regression analysis model showed a well-fitted model, in which NLEs and alexithymia explain a total of 34.2% of the variance of depressive symptoms in these participants. NLEs (*β* = 0.256, *p* < 0.001) and dimensions of alexithymia (difficult describing feelings (*β* = 0.192, *p* < 0.001) and identifying feelings (*β* = 0.308, *p* < 0.001)) were positively correlated with symptoms of depression.

**Conclusions:**

Previous studies have confirmed the correlation between NLEs and depression, alexithymia and depression, respectively. In our study, we used a stepwise regression model to explain the relationship among those variables simultaneously, and found that NLEs and alexithymia could function as predictors of depressive symptoms. Based on this discovery, alexithymia-focused treatment strategies could be alternative in depressive patients with alexithymia, but this remains to be verified in the future.

**Supplementary Information:**

The online version contains supplementary material available at 10.1186/s12888-024-05902-0.

## Introduction

Depression is common, possibly severe and life threatening, and is one of the leading contributors to the Global Burden of Disease [1]. In any 12-month time period, an episode of depression could happen in about 4.7% (4.4–5.0, 95% *CI*) of the world’s population [2]. One priority is the prevention [3] and early intervention [4] of depression, which means an intervention just before the structural brain changes become more evident with recurrence [5, 6]. Thus, not only the symptomatic treatment of depressive symptoms, but also the identification and intervention of predisposing factors and antecedents are equally important [7].

Multiple factors, including biological factors (e.g., genetic, endocrine, or inflammation factors), various precipitating (e.g., stressful life events) and perpetuating factors (e.g., substance use, behavioral patterns), are likely to operate in the development of depression [8]. A widely accepted model to incorporate all these factors is diathesis-stress model (which is also named as vulnerability-stress model) [8]. This model illustrates that with a higher degree of vulnerability, a person could become depressed even with a mild stressor [9].

Negative life events (NLEs), such as health problems and interpersonal strain, functioning as stressor, can prompt the development of depression [10]. Depressed patients reported more NLEs than control group [11]. Previous findings confirmed significant and positive associations between NLEs and the onset, recurrence and persistence of depressive symptoms [12]. However, individuals exposed to NLEs may not necessarily have depressive symptoms. Based on diathesis-stress model, individual differences can affect their perception and response to life events, as well as their assessment of stressors [9]. Susceptible individuals are more likely to suffer from depression through some physical qualities (e.g., genetic predispositions) or psychological qualities (e.g., personality traits) [13] under the influence of NLEs. Therefore, it is necessary to find which kind of diathesis could influence depressive symptoms in the case of NLEs, so that such factors could then be managed effectively in prevention or treatment efforts.

Alexithymia is a deficit in the cognitive processing of emotions. It is characterized by difficulties in identifying feelings, describing feelings, externally oriented thinking style, and limited daydreaming [14]. According to the stress-alexithymia hypothesis [15], individuals with alexithymia tend to make negative and exaggerated assessments of the environment due to difficulties in emotion recognition and description, and have reduced ability to deal with stress. This improper assessment of challenges and threats of the external environment eventually led to individuals with alexithymia in a state of stress [16]. The interaction between alexithymia and stress perception can prompt the development of depression [17]. Studies have confirmed the significant and positive correlation between alexithymia and depression both in clinical [18] and community samples [19]. The depressive symptoms of patients with different levels of alexithymia were significantly different [18–20]. Alexithymia is a predictor of developing depression, and reducing alexithymia can alleviate depressive symptoms to a certain degree [21].

Although the correlations between the investigated variables have been well established respectively, the internal mechanisms among NLEs, alexithymia, and depressive symptoms have not been fully explored. This study intends to: (1) explore the relationship among NLEs, alexithymia, and depression symptoms, and (2) to express this relationship by a regression model based on the outpatient sample in China.

## Methods

### Participants

All patients visiting the outpatient clinic of the department of psychological medicine, Peking Union Medical College Hospital during the time interval from June, 2020 to April, 2023 who completed the set of following described questionnaires were selected. The main types of diagnosis in our clinic include anxiety disorders, depressive disorders, somatic symptom disorders, insomnia, and etc. And if the patients visited our clinic more than once, we only selected the result of questionnaires at their first visit.

The study design was approved by the Ethics Committees of Peking Union Medical College Hospital. The investigation was carried out in accordance with the latest version of the Declaration of Helsinki. And informed consent of the participants was obtained before the survey.

### Measurements

#### Negative life events

Negative life events (NLEs) were measured by the Life Events Scale (LES), which is a self-report questionnaire containing 48 items and has been widely used in Chinese population [22, 23]. It is used to measure perceived stressfulness and number of stressful life events in terms of three dimensions: family life (28 items); work and study (13 items); social and other aspects (7 items). It captures NLEs including bereavement, serious illness, unemployment, financial crises, and social difficulties. Each life event is evaluated on the following five aspects: nature of the event, the time of occurrence, the severity of impact, the duration of impact, and the number of occurrences of the event. The score of the perceived stress is multiplied by the last three aspects. Higher scores for NLEs indicate greater stress perceived [24]. The Cronbach alpha value of this scale in the current study is 0.82.

#### Depression

Depressive symptoms were assessed using the 9-item Patient Health Questionnaire (PHQ-9) [25]. PHQ-9 scale was developed to describe the severity of depressive symptoms within the prior two weeks. A meta-analysis summarized the psychometric properties of PHQ-9 for the depression screening in different medical settings and concluded that PHQ-9 is an instrument which suitable for a range of departments, countries, and populations [26]. This scale consists of 9 items with a four-point Likert scale from 0 (not at all) to 3 (nearly every day). The composite score is 0 to 27. Higher score indicates higher level of depression. Regarding severity, the score of PHQ-9 is divided into five grades: no depressive symptoms (0–4); mild (5–9); moderate (10–14); moderately-severe (15–19); severe (20–27). The optimal cutoff value for PHQ-9 to identify positive cases varies from study to study, with the most common recommended cutoff value being 10 points [27]. The Chinese version of the PHQ-9 has been verified to have good reliability and validity [28]. The Cronbach alpha value of this scale in the current study is 0.89.

#### Alexithymia

Alexithymia was assessed by the Toronto Alexithymia Scale (TAS-26) [29], which contains 26 self-descriptive statements, and has demonstrated good reliability and validity across a range of samples [30]. It is a 5-point Likert scale, each item rated from 1 (strongly disagree) to 5 (strongly agree). The total score is between 26 and 130 points. Higher scores indicate a higher level of alexithymia. The TAS-26 consists of 4 subscales, three of them were used in our present study. They include: (1) difficulty describing feelings to others (DDF), (2) difficulty identifying feelings (DIF), and (3) externally oriented thinking (EOT). The test authors Taylor et al. [31] reported that the last factor (limited daydreaming) lacked correlation with the other three. Thus, the scale score calculated in our present study is the sum of the first three factors. The Cronbach alpha value of this scale in the current study is 0.83.

### Data analysis

Continuous variables are presented as means and SD; categorical variables are presented as percentages. The Pearson correlation coefficient was used to test the correlation among NLEs, alexithymia and depressive symptoms. To further investigate the relationship between the research variables, NLEs and alexithymia were set as specific predictor variables and the severity of depressive symptoms was set as the dependent variable to establish linear regression analyses after adjusting for gender and age of subjects. Stepwise regression was used to select the sub-scales that were highly associated with the depressive symptoms, and determine a mathematical expression that represented the relationship between depressive symptoms, NLEs and alexithymia. The statistical analyses were performed with IBM SPSS Statistics 25.0 and statistical significance for all analyses was set to *p* < 0.05 (two-tailed).

## Results

### Participant characteristics

In this study, a total of 2747 outpatients were recruited, among them, 762 (27.7%) were males and 1985 (72.3%) were females. The age of participants ranged from 18 to 65 years (*M* = 34.99, *SD* = 9.54). The total score of PHQ-9 ranged from 0 to 27 with a mean of 13.05 (*SD* = 6.39). Among them, 1840 (67.0%) had a PHQ-9 score of ten or higher. The mean score of the NLEs was 112.06 (*SD* = 126.40). The mean score of the TAS was 57.81 (*SD* = 10.68) (Table 1). There was no statistically difference in the scores of LES-Negative Life Events (113.18 vs. 105.17, *P* = 0.227), total TAS (57.77 vs. 58.03, *P* = 0.640) and PHQ-9 (13.00 vs. 13.32 *P* = 0.335) between the patients visiting our clinic before December 7, 2022 and after, in which COVID-19 restrictions in China have been fully lifted.

The distribution of diagnoses among the sampled population and the average scores of questionnaires in each diagnosis were detailed in Supplementary Table 1. The mean score for patients with a diagnosis of depression disorders was significantly higher than patients with others in each scale (*P* < 0.01). Moreover, in depression patients, it was the first time for most of them (89.5%) to receive psychological counseling on their psychological problems. No difference was found between the patients who had been treated with any kind of therapies before and those who had not in the measurement of NLEs, alexithymia or severity in depression symptoms.

**Table 1:** Sample characteristics (*n* = 2747)

	Total outpatients (*n* = 2747)
Gender (female, %)	72.3 (*n* = 1985)
Age (*M* ± *SD*)	34.99 ± 9.54
PHQ-9 total score (*M* ± *SD*)	13.05 ± 6.39
No depressive symptoms (0–4, %)	8.7
Mild depressive symptoms (5–9, %)	24.3
Moderate depressive symptoms (10–14, %)	10.4
Moderately-severe depressive symptoms (15–19, %)	38.6
Severe depressive symptoms (20–27, %)	18.0
PHQ-9 cut-off ≥ 10 (%)	67.0
LES-Negative Life Events total score (*M* ± *SD*)	112.06 ± 126.40
TAS-Difficulty in Describing Feelings score (*M* ± *SD*)	17.17 ± 5.21
TAS-Difficulty in Identifying Feelings score (*M* ± *SD*)	22.10 ± 5.19
TAS-Externally Oriented Thinking score (*M* ± *SD*)	18.54 ± 3.67
TAS total score (*M* ± *SD*)	57.81 ± 10.68

Note: LES: Life Events Scale; PHQ-9: 9-item Patient Health Questionnaire; TAS: Toronto Alexithymia Scale; M: mean

### Correlation between clinical variables

Pearson correlation analysis showed that all the three variables were significantly correlated with each other (*p* < 0.05) (Supplementary Table 2).

The PHQ-9 score was significantly and positively correlated with NLEs, difficulty in describing feelings and difficulty in identifying feelings (*p* < 0.05), but not correlated with externally oriented thinking (Supplementary Table 3).

### Linear regression analyses

The results showed that NLEs, alexithymia significantly predicted the depressive symptoms after adjustment of age and gender (*p* < 0.01). NLEs explain 20% of the variations in depression symptoms. Alexithymia explains 26% of the variations in depression symptoms (Table 2).

**Table 2:** Linear regression model with negative life events, alexithymia as predictor variables predicting depressive symptoms

Dependent Variables	Predictors	B	β	t	*R* ^2^	F
Depressive Symptoms	Negative Life Events	0.019	0.385	22.391	0.200	228.993^**^
	Alexithymia	0.277	0.462	27.829	0.262	325.241^**^

Note: *B*: parameter estimate; *β*: standardized estimate; ^**^p < 0.01

### Stepwise regression analyses

The subscale of EOT did not reach statistical significance and was excluded in the stepwise regression analysis model. NLEs, difficulty in describing feelings and identifying feelings were positively correlated with depression symptoms (*p* < 0.001) (Table 3). And a linear distribution was described by [PHQ-9] = -0.843 + 0.013 × [LES-NLEs] + 0.236× [TAS-DDF] + 0.380 × [TAS-DIF] (*p* < 0.001).

The sum of the squared multiple correlations (R^2^) for the variable of depressive symptoms is 0.342, which indicates that NLEs and alexithymia explain a total of 34.2% of the variance of depressive symptoms in these participants.

**Table 3:** Stepwise regression results for depression symptoms and variables

Predictors	B	β	t	*p*	VIF	R2	F
Negative Life Events	0.013	0.256	16.088	< 0.001	1.057	0.342	474.968, *p* < 0.001
TAS-1	0.236	0.192	8.157	< 0.001	2.312		
TAS-2	0.380	0.308	12.970	< 0.001	2.354		

Note. *B*: parameter estimate; *β*: standardized estimate; TAS-1: Difficulty in Describing Feelings; TAS-2: TAS-Difficulty in Identifying Feelings

## Discussion

### General characteristics of our sample

Our study enrolled 2747 outpatients from a psychosomatic outpatient sample in China. Most of the patients were counselling our clinic with a final diagnosis of anxiety disorder (45.8%) or depressive disorder (40.2%), corresponding with the epidemiological data of mental disorders in China [32]. Patients with a diagnosis of depression disorders appeared to have a worse outcome of the scale assessment than the patients with other disorders, demonstrating that NLEs and alexithymia could have a more significant influence in patients with depression than other mental diseases. In our study, for most of the patients, it was the first time to receive psychological counseling or medical treatments on their psychological symptoms. However, no difference was observed between the treated and untreated patients. We speculate that, on the one hand, the result is limited by the small sample size of patients in therapy. And on the other hand, the patients at their first visit to our clinic with a previous medical history could have a poor medical adherence or receive non-standardized therapy strategies, resulting in no differences of any measurements between groups.

Our measures were collected during the COVID-19 pandemic, which is a global public health event. Previous studies have demonstrated that the epidemiological burden [33–35] and severity [36] of post-COVID-19 depressive symptoms obviously exceed the pre-pandemic ones. Negative life events [37] and alexithymia [38] are also impacted by the pandemic of COVID-19. We tried to compare the results of measurements before and after the fully lifting of the restriction in China, but found no difference between them. We suppose that it could be a little rough to divide the data just at a specific time point in current study. A period of time remains to be spent in the appearance of aftereffects of the policy.

### NLEs and depressive symptoms

Consistent with previous studies [39], the present study found that NLEs were associated with depressive symptoms and more NLEs were associated with higher severity of depression. NLEs are one of the most studied psychosocial factors that precipitate the development and maintenance of depressive symptoms [40, 41]. NLEs may also trigger other psychological disorders and even some life-threatening behaviors [42]. There is much evidence showing that NLEs in the past lives (childhood or adolescence) have considerable effects on the mental and physical health of adults [43]. NLEs can increase the emotional burden of individuals in the form of stressfulness, and ultimately correlating with the depression [44].

### Alexithymia and depressive symptoms

26% of the variation in depressive symptoms could be explained by alexithymia in our study. Many studies have previously identified that alexithymia played a role in the development of depression [21] and some other conditions. In the study by Li et al. [45] with 2421 parents, alexithymia played a significant role on parenting-related mental health. Radetzki et al. [46] found that alexithymia played a mediating role in the association of insecure attachment and the illness severity of depression and social anxiety. The level of alexithymia, especially difficulty in describing feelings to others, is higher in individuals with depression than in healthy controls [47]. And more severe depressive symptoms are associated with more severe alexithymia [46].

In addition, when referring to the result of Pearson correlation analysis and stepwise regression model, we found that the subscale of DDF and DIF were correlated with PHQ-9 with a great significance, while EOT was not. Previous studies [48] have also demonstrated that EOT was not correlated with depression severity. EOT is reported to be associated with the visualization and process of negative images [49]. For those with defect in EOT, it is speculated that with a segregation of negative emotions, a protective role could be played in the management of stress or NLEs in a short period [38, 50], which could result in a poor correlation with depression severity.

### NLEs, alexithymia, and depression symptoms

Previous study and our study have demonstrated the association between depressive symptoms and NLEs [39], depressive symptoms and alexithymia [21], respectively. However, only 20%, and 26% variations in depressive symptoms were explained by NLEs, and alexithymia (Table 2). On top of this, result of Pearson correlation analysis also demonstrated that NLEs and alexithymia were related to each other. Tychey et al. [51]. reported that stressful life events in early perinatal can contribute to alexithymia. Alexithymia may represent a learned pathway in order to escape from unpleasant affect [52]. NLEs could be an important factor that enhances the development of alexithymia by disrupting normal emotions [53, 54].

Therefore, taking the factors illustrated above into consideration, we wondered whether incorporating the two variables could make a more valid prediction of the depressive symptoms. By including the two variables, or more specifically, the subscales of the two variables, into the stepwise regression analysis, the regression model could make a better prediction to a certain degree, which could explain 34.2% variations in the symptoms of depression. Following the diathesis-stress model, we speculated that with a higher degree of alexithymia, a person could develop depressive symptoms more easily when exposing to NLEs. Previous stress-alexithymia hypothesis has suggested that individuals with alexithymia are more susceptible to stress through the interaction of three primary components: a lack of ability to identify emotions (cognitive component), a lack of ability to express and process emotions, and a tendency to exaggerate the somatovisceral response (physiological component) [15]. In response to NLEs, individuals with alexithymia are prone to percept excessive stressfulness coupled with poor ability to deal with stress, which could ultimately contribute to depression [16, 17].

Based on the discovery of our study, in order to alleviate the depressive symptoms, alexithymia could be a promising direction, when compared with NLEs, which represent a kind of unpredictable conditions. It has been widely discussed before whether alexithymia is a state dependent state or a stable strait [55]. And the growing consensus is that alexithymia is a trait that can be modified through therapy, although the exact extent to which alexithymia can be improved varies from person to person [56, 57]. According to the result of our study, therapy strategies proposed before for the improvement of alexithymia could be potentially valuable in the alleviation of depressive symptoms, such as emotion-focused therapies like mindfulness based [57, 58] and emotional psychoeducation interventions [56].

In summary, we find that there is a close relatedness among NLEs, alexithymia, and depression. Negative life events and alexithymia correlate with depressive symptom and explain a good bit of the variance. A stepwise regression analysis shows that depression symptoms could be predicted by both alexithymia and NLEs to a certain degree. Based on this discovery, alexithymia-focused treatment strategies could be an alternative approach in the treatment of depressive patients with alexithymia. However, it still remains to be verified in the future.

## Limitations

There are several limitations in present study. Firstly, we used self-reported questionnaires in the survey, and as a result, recalling bias and confounding factors like educational levels and comprehension ability may exist. Secondly, this study only evaluates the correlation among cross-sectional measures, which is insufficient to verify the causal relationships between NLEs, alexithymia, and depressive symptoms. In our study, we selected NLEs and alexithymia as the predictors, and depressive symptoms as the outcome to construct our hypothesis based on previous study. However, other causal patterns are also possible (for example, one in which depressive symptoms predict alexithymia and/or negative life events). Additional longitudinal empirical investigations should be conducted in the future, like whether making an intervention on alexithymia could alleviate the depressive symptoms. Finally, we only adjusted age and gender. It would be much better to collect more social demographic (e.g. socioeconomic status, nationality) and biological factors (e.g. complications, smoking, drug abuse, family history) at baseline and make a more comprehensive evaluation.

## Conclusion

We firstly conducted a survey in China exploring the relationship among NLEs, alexithymia, and depressive symptoms. We used a stepwise regression model to explain the relationship among those variables simultaneously, and found that NLEs and alexithymia could function as predictors of depressive symptoms. Alexithymia-focused treatment strategies could be alternative in depressive patients with alexithymia but this remains to be verified in the future.

### Electronic supplementary material

Below is the link to the electronic supplementary material.

Supplementary Material 1

## Data Availability

The data that support the findings of this study are available from the authors but restrictions apply to the availability of these data, which were used under license from the Peking Union Medical College Hospital for the current study, and so are not publicly available. Data are, however, available from the authors upon reasonable request and with permission from the Peking Union Medical College Hospital.

## Declarations

### Statement

The paper has not been previously published. Authors of this article had access to all study data, are responsible for all contents of the article, and had authority over manuscript preparation and the decision to submit the manuscript for publication. All listed authors have approved of the submission of the manuscript to the journal.

## Abbreviations

LES: Life Events Scale
NLEs: negative life events
TAS-26: Toronto alexithymia scale
DDF: difficulty describing feelings
DIF: difficulty identifying feelings
EOT: externally oriented thinking
PHQ-9: 9-item Patient Health Questionnaire
