# Supplementary material for: The association among negative life events, alexithymia, and depressive symptoms in a psychosomatic outpatient sample
Source: BMC Psychiatry. 2024 Jun 18;24:451. doi: 10.1186/s12888-024-05902-0 (PMC11186062; doi:10.1186/s12888-024-05902-0)
Supplement: Supplementary file 1 — Supplementary Material 1 [file 12888_2024_5902_MOESM1_ESM.docx]

**Supplementary Table 1**

**The distribution of diagnoses among the sampled population (n = 2747).**

| Diagnosis* | Participants, n (%) | LES-Negative Life Events total score (*M* ± *SD*) | TAS total score  (*M* ± *SD*) | PHQ-9 total score  (*M* ± *SD*) |
| --- | --- | --- | --- | --- |
| anxiety disorders | 1259 (45.8%) | 92.74 ± 114.58 | 55.53 ±10.19 | 11.01 ± 5.88 |
| **depression disorders** | **1105 (40.2%)** | **141.24 ± 137.67** | **60.92 ±10.60** | **15.96 ± 5.88** |
| obsessive-compulsive disorders | 76 (2.8%) | 84.01 ± 89.98 | 56.82 ± 8.93 | 10.96 ± 5.57 |
| somatic symptom disorders | 72 (2.6%) | 100.94 ± 154.47 | 55.31 ± 10.39 | 10.32 ± 5.71 |
| insomnia | 57 (2.1%) | 93.40 ± 114.71 | 56.30 ± 10.80 | 11.84 ± 5.71 |
| others (eg. bipolar disorder, eating disorders) | 178 (6.5%) | 87.86 ± 99.20 | 56.46 ± 10.54 | 11.79 ± 6.68 |

*For patients with more than on diagnoses, only first diagnosis was included.

Note. LES: Life Events Scale; PHQ-9: 9-item Patient Health Questionnaire; TAS: Toronto Alexithymia Scale; M: mean.

**Supplementary Table 2**

**Correlations between negative life events, alexithymia and depressive symptoms (n = 2747).**

| Variables | 1 | 2 | 3 |
| --- | --- | --- | --- |
| 1. Negative Life Events | 1 |  |  |
| 2. Alexithymia | 0.190^**^ | 1 |  |
| 3. Depressive Symptoms | 0.364^**^ | 0.480^**^ | 1 |

^**^*p* < 0.01.

**Supplementary Table 3**

**Correlations among study variables (n=2747).**

| Variables | 1 | 2 | 3 | 4 | 5 |
| --- | --- | --- | --- | --- | --- |
| 1. Negative Life Events | 1 |  |  |  |  |
| 2. Difficulty in Describing Feelings | 0.190^**^ | 1 |  |  |  |
| 3. Difficulty in Identifying Feelings | 0.231^**^ | 0.753^**^ | 1 |  |  |
| 4. Externally Oriented Thinking | -0.044^*^ | 0.114^**^ | 0.037 | 1 |  |
| 5. Depressive Symptoms | 0.364^**^ | 0.473^**^ | 0.512^**^ | 0.002 | 1 |

^*^*p* < 0.05, ^**^*p* < 0.01.
